# A typical case of resected pancreatic hamartoma: a case report and literature review on imaging and pathology

**DOI:** 10.1186/s40792-020-00869-y

**Published:** 2020-05-24

**Authors:** Hokahiro Katayama, Kyohei Azuma, Kenji Koneri, Makoto Murakami, Yasuo Hirono, Satomi Hatta, Yoshiaki Imamura, Takanori Goi

**Affiliations:** 1 1st Department of Surgery, University of Fukui, 23-3 Matsuoka Shimoaiduki, Eiheiji-cho, Yoshida-gun, Fukui, Japan; 2 Department of Pathology, University of Fukui, 23-3 Matsuoka Shimoaiduki, Eiheiji-cho, Yoshida-gun, Fukui, Japan

**Keywords:** Pancreatic hamartoma, Characteristics, Imaging, Pathology

## Abstract

**Background:**

Pancreatic hamartomas are rare entities and difficult to diagnose before resection. We report a case of resected pancreatic hamartoma and literature review of typical characteristics of the lesion.

**Case presentation:**

A 78-year-old man presented with a mass in his pancreas, which was incidentally identified when he experienced pneumonia. No remarkable symptoms were observed, and laboratory tests showed no abnormalities, except a slight carcinoembryonic antigen elevation. Enhanced computed tomography and magnetic resonance imaging showed a well-demarcated solid mass with heterogeneous contrast that was 2 cm in size. A gradual enhancement pattern was also observed. The biopsy revealed no specific findings; therefore, surgical resection was necessitated to confirm the diagnosis. Histopathologically, ducts, acinar cells, and adipose cells without atypia were observed among abundant fibrous stroma, but islets of Langerhans and peripheral nerves were absent. An immunohistochemical examination demonstrated CD34 and c-kit positive staining in the stromal cells, S-100 positivity in the adipose cells, and a lack of elastic fibers in the duct walls. The lesion was diagnosed as a pancreatic hamartoma.

**Conclusion:**

Asymptomatic pancreatic hamartomas can avoid resection. A careful consideration of imaging and appropriate immunohistochemistry of biopsy specimen may facilitate accurate diagnosis before resection. Therefore, sufficient recognition of the characteristics of pancreatic hamartomas is desirable.

## Background

Pancreatic hamartomas are benign lesions, rather than tumors, that are composed of mature cells with malformed structures. These lesions are extremely rare, and no studies thus far have reported pre-operative diagnoses. Instead, pancreatic hamartomas are often diagnosed as other pancreatic tumors that require surgical resection for a definitive diagnosis. We report a case of a pancreatic hamartoma and review the related literature to clarify the clinical, radiological, and pathological characteristics of this lesion.

## Case presentation

**Fig. 1:**
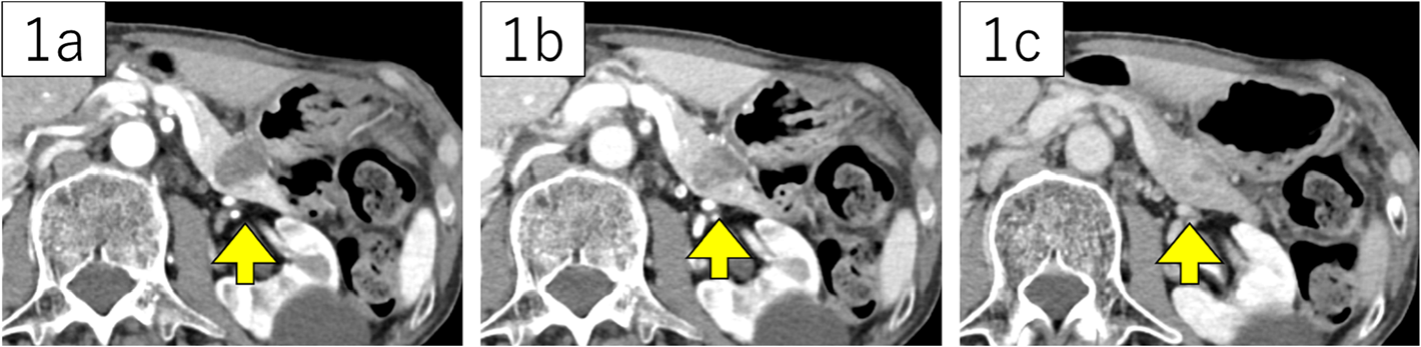
Dynamic computed tomography (CT) showed hypodensity in the arterial phase (**1a**), slight enhancement in the portal phase and iso- to hyper-density in the equilibrium phase (**1b**), compared to the parenchyma of the pancreas (**1c**), each indicated with arrowheads

**Fig. 2:**
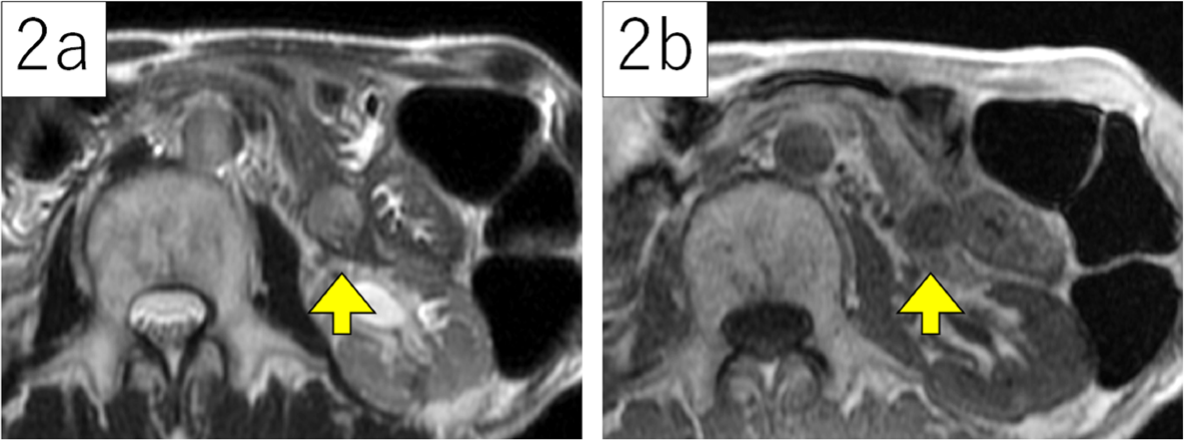
Magnetic resonance imaging (MRI) showed high signal intensity in T2WI (**2a**) and low intensity in T1WI (**2b**), each indicated with arrowheads

**Fig. 3:**
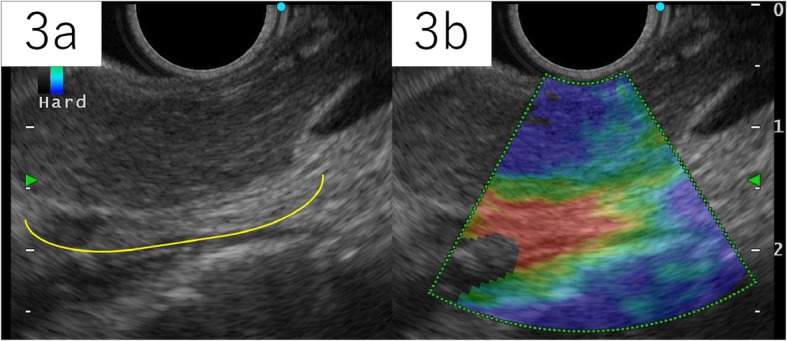
Endoscopic ultrasonography (EUS) depicted well-demarcated mass (indicated with arched line) with inside mosaic pattern (**3a**), and the mass was described harder than the parenchyma of the pancreas by elastography (**3b**)

A 78-year-old man was referred to our hospital for a tumor-suspected lesion in his pancreatic tail that was detected by computed tomography (CT) when he was treated for pneumonia. He repeatedly experienced pneumonia, but his other prior illnesses and family history were unremarkable. He was asymptomatic, and his laboratory examination showed almost normal results, including amylase levels, except elevated carcinoembryonic antigen (CEA) levels of 6.4 ng/mL. Carbonhydrate antigen 19-9 (CA19-9) and pancreatic cancer-associated antigen-2 (DUPAN-2) levels were within normal ranges. CT revealed a 22-mm mass in his pancreatic tail, without dilatation of the main pancreatic duct. Retrospectively, the lesion was found to be slightly smaller in the CT image that was taken one and a half years ago to evaluate pneumonia. On dynamic enhancement, the lesion manifested an increased late enhancement pattern compared to the parenchyma of the pancreas, showing hypodensity with well-demarcation in the arterial phase, slight enhancement from the marginal area in the portal phase, and iso- to hyper-density with heterogeneous contrast in the equilibrium phase (Fig. 1). Magnetic resonance imaging (MRI) acquired a T2-weighed image (T2WI) with high signal intensity and a T1-weighed image (T1WI) with low intensity (Fig. 2), and no cholangial structural abnormalities were observed. Endoscopic ultrasonography (EUS) depicted a mosaic pattern inside the lesion, and EUS-elastography showed the components of the mass harder compared with the parenchyma of the pancreas (Fig. 3). No uptake into the lesion was observed on 18F-fluorodeoxyglucose-positron emission tomography (FDG-PET). With the above findings, we considered the possibility of acinar cell carcinoma, neuroendocrine tumor (NET), and solid and pseudopapillary neoplasm (SPN), each with atypical features. The specimen acquired by fine-needle aspiration under EUS (EUS-FNA) presented almost normal tissue. However, we could not clearly deny the tumors which require resection; therefore, a distal pancreatectomy was performed.

**Fig. 4:**
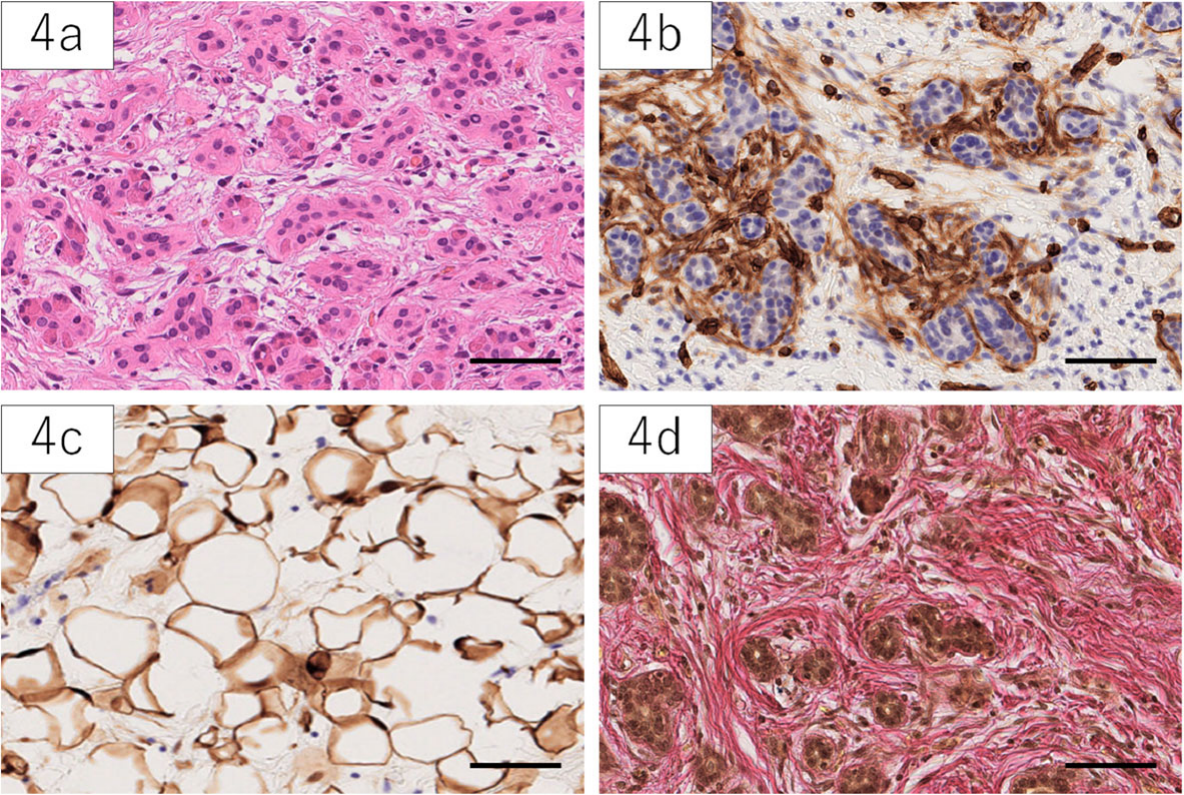
HE staining showed small ducts within abundant fibrous stroma (**4a**). The spindle-like stromal cells expressed CD34 (**4b**). Adipose tissue showed expression of S-100 (**4c**). Elastica van Gieson staining indicated the absence of elastic fibers around the ducts (**4d**). Scale bar 100 μm

The surgical specimen showed a soft elastic lesion with a smooth surface that did not invade into the surrounding tissue. The cut surface exhibited a slightly reddish solid component, with a small internal adipose content. The background of the pancreas did not seem to have chronic pancreatitis. Microscopically, small ducts, acinar structures, and adipose tissue were observed within the abundant fibrous stroma, but islets of Langerhans were not detected. In addition, peripheral nerves within the lesion were absent. Immunohistochemistry (Fig. 4) showed positive staining for CD34, c-kit, and ER in the stromal cells; S-100 positivity in adipose cells; and CK7, CK20, and BCL-2 positivity in duct cells. The MIB index was below 1%. Elastica van Gieson staining showed an absence of elastic fibers around the ducts.

## Discussion

In 1904, Albrecht described hamartomas as a tumor-like malformation [1] that is composed of normal tissue elements of the organ of the origin, but show abnormalities in the amount, ratio, or distribution of the tissue elements. Hamartomas commonly develop sporadically, but are also seen in genetically disordered syndromes, such as tuberous sclerosis or PTEN hamartoma tumor syndrome (PHTS) [2]. Hamartomas have the potential to be derived from every anatomical sites, and many organs, such as the spleen, liver, kidneys, and lungs, have been reported to be the origins [3]. Their imaging characteristics and pathologies vary depending on the organ; therefore, diagnoses have to be made uniquely. Pancreatic hamartomas are rare, and their accumulative characterization has been insufficient, which makes their diagnosis difficult.

Patients with pancreatic hamartomas usually present without specific symptoms, and the lesions are found incidentally by imaging examinations in many of the cases. Recently, the number of case reports about pancreatic hamartomas has been gradually increasing, partially due to the progress in quality and accessibility of imaging examinations and due to the widespread acknowledgment of the entity. With the accumulation of only 40 reported cases to date [4–27], including our case, the clinical and pathological features are becoming clarified (Table 1).

**Table 1:**
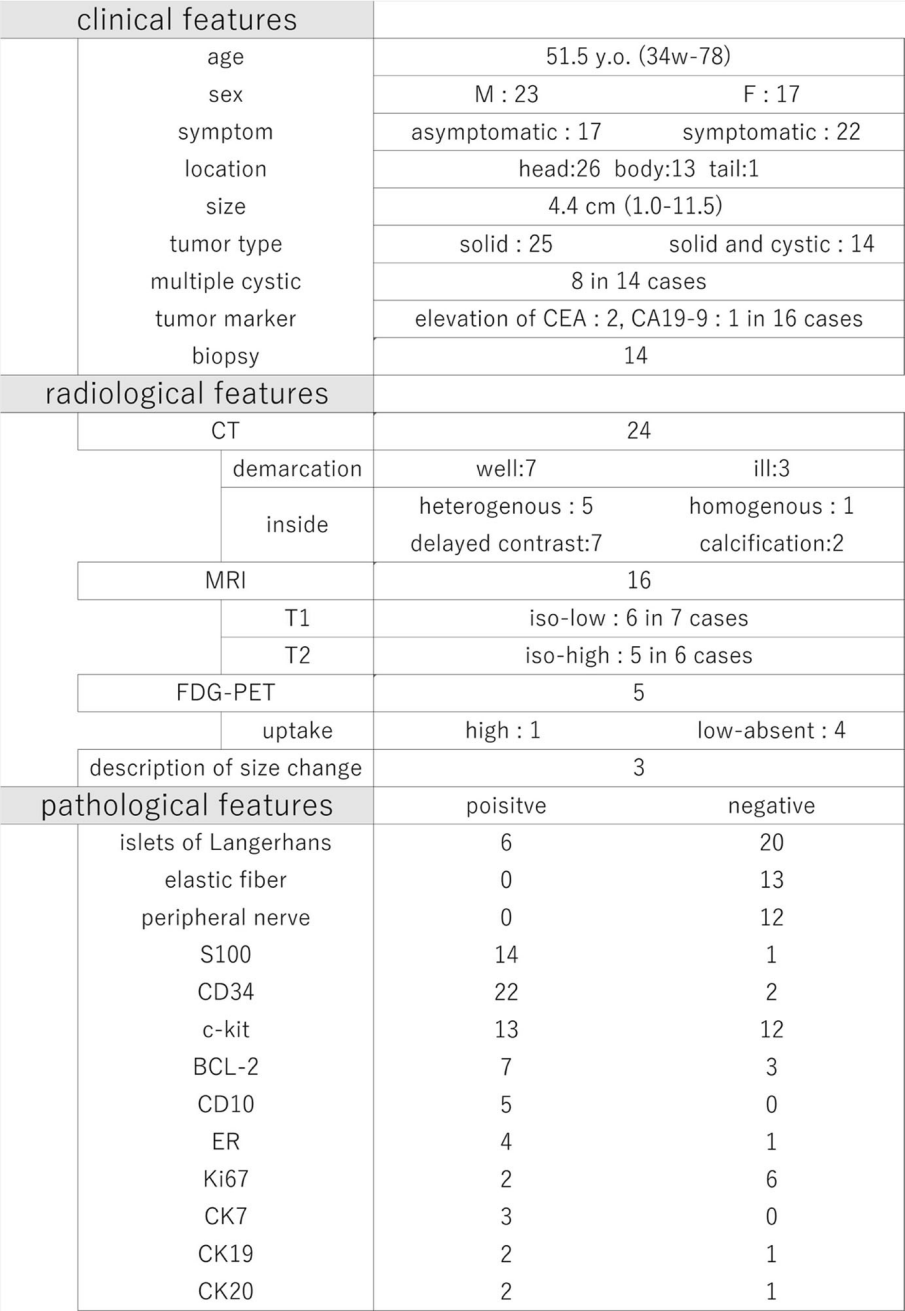
Summarized clinical, radiological, and pathological features of reported pancreatic hamartomas in the literature

The mean age of patients with pancreatic hamartomas was 51.5 years (34 weeks–78 years), there was no apparent difference in sex, and the male-to-female ratio was 1:0.78. Forty-four percent of patients were asymptomatic at the time of detection. Tumor markers were measured in 16 cases, while CEA elevations were only observed in 2 cases, and CA19-9 elevation was found in 1 case. Biopsies were performed in 14 cases, none of which detected malignant tissue nor contributed to an accurate diagnosis. Morphologically, the mean size was 4.4 cm (1.0–11.5 cm), and the tumors were mainly localized to the pancreatic head (65%). A solid pattern was seen in 64% of patients, while a solid and cystic pattern in 36%. Among the latter, multi-cystic lesions were observed in 57% (8/14).

Among the literature, imaging examination findings have been reported in 26 cases, CT in 24, and MRI in 16 cases. CT and MRI described the lesions as well-demarcated in 70% of patients and internally heterogeneous in 83%. MRI depicted the solid component as an iso-low intensity on T1WI in 86% and as iso-high on T2WI in 83% of cases. Enhanced CT or MRI showed late enhancement patterns in all seven patients, which presented as lower contrast in the early phase and iso-high contrast in the late phase compared with the parenchyma of the pancreas. FDG-PET demonstrated uptake in 20% of lesions, which may have misled us into diagnosing the lesions as pancreatic carcinoma. NETs are usually homogenous and show high-contrast patterns in the early phase of enhancement, although they sometimes present low-contrast and heterogeneous images that result from the degeneration or necrosis when the size becomes large [28]. SPNs are uniquely accompanied by bleeding or calcification, both of which are seen in less than 30% of cases [29]. Pancreatic ductal adenocarcinoma resembles pancreatic hamartomas in several aspects. However, if the lesion remains stable over some period, such as in our case, the possibility of adenocarcinoma is considered low. Pancreatic hamartomas exhibit confusable image with the above tumors that usually require resection, but the image is not typical for each tumor. Therefore, although it may be difficult to make an accurate diagnosis with only images, it is important to suspect pancreatic hamartoma as a possible disease and compare these features.

Histopathologically, the lesion usually contains mature ducts and acinar cells that are embedded in the abundant fibrous stroma. The structures of the ducts and acini show distorted architectures that do not exhibit atypia. Yamaguchi [13] pointed out the absence of elastic fibers in duct walls, peripheral nerves, and islets of Langerhans as a unique triad of pancreatic hamartoma. In the literature, the absence of elastic fibers has been reported in 100% of cases (13/13), the absence of islets in 77% (20/26), and the absence of peripheral nerves in 100% (12/12), indicating highly specific findings. Duct cells or adipose cells have been found to exhibit S-100 positivity in 93% (14/15) of patients, which is usually a marker for endocrine cells in the normal pancreas [30]. Stromal cells in 91.7% (22/24) of cases showed CD34 positivity, but this can also be detected in the stroma of chronic pancreatitis, which complicates the interpretation of biopsy results. Because c-kit was only detected in 52% (13/25) of patients and can be expressed in other tumors [31], the utility of c-kit positivity is not considered high. Although with limited reports, positive bcl-2 (70%, 7/10), positive CD10 (100%, 5/5), positive ER (80%, 4/5), and negative Ki67 (75%, 6/8) can be supportive diagnostic factors. CD34 and c-kit positivity is also suggestive of GISTs, but histologically, detection of pancreatic endocrine and exocrine components inside the mass can lead to negative diagnosis for GISTs. Furthermore, DOG1 (discovered on GIST-1) is suggested to be a negative marker of pancreatic hamartomas [5]; therefore, additional staining with DOG1 can be useful to distinguish hamartomas from GISTs.

Pancreatic hamartoma is not a neoplasm; therefore, surgical resection should be avoided if the lesion is not accompanied by related symptoms. All prior reports have described a definitive diagnosis after resection, but in asymptomatic patients who comprise of almost half of the cases, unnecessary surgery may have been avoided if hamartoma was strongly suspected. Especially, the case we experienced possessed a typical clinicopathological features; therefore, careful consideration of results of examinations might have led to the accurate diagnosis. If the findings of imaging examination were suggestive of pancreatic hamartoma, the identification of the absence of elastic fibers in the duct wall, peripheral nerves, and islets, as well as staining for S-100 and CD34, with EUS-FNA are definitively helpful.

## Conclusion

Pancreatic hamartomas are extremely rare entities and regarded to be difficult to be diagnosed preoperatively. But it is suggested that appropriate diagnosis and treatment strategy can be established if the examinations are interpreted with respect to the possibility of a pancreatic hamartoma. Therefore, the widespread and sufficient recognition of the disease is desirable.

## Data Availability

All data generated or analyzed in this study are included in this manuscript, but available from the corresponding author on reasonable request.

## Abbreviations

CT: Computed tomography
CEA: Carcinoembryonic antigen
CA19-9: Carbonhydrate antigen 19-9
DUPAN-2: Pancreatic cancer-associated antigen-2
MRI: Magnetic resonance imaging
T2WI: T2-weighed image
T1WI: T1-weighed image
EUS: Endoscopic ultrasonography
FDG-PET: 18F-Fluorodeoxyglucose-positron emission tomography
NET: Neuroendocrine tumor
SPN: Solid and pseudopapillary neoplasm
FNA: Fine-needle aspiration
